# Genome-wide association analysis was used to discover genes related to soybean grain weight per plant and 100-grain weight

**DOI:** 10.1270/jsbbs.23057

**Published:** 2024-06-26

**Authors:** Tingting Sun, Qi Zhang, Lu Liu, Yujie Tang, Jiabao Wang, Kun Wang, Boran Yuan, Piwu Wang

**Affiliations:** 1 Jilin Agricultural University, Changchun 130000, China; 2 Jilin Academy of Agricultural Sciences, Changchun 130000, China

**Keywords:** soybean, GWAS, 100-grain weight, grain weight per plant

## Abstract

As an essential grain, oil, and feed crop worldwide, soybean plays a crucial role. Developing high-yielding and high-quality soybean varieties is a critical goal for breeders. The grain weight per plant and 100-grain weight directly impact the soybean yield. This study combined genotypic data from the population with phenotypic data. Based on genome-wide association analysis (GWAS), GLM and MLM analysis models were used to locate the Gm04_21489088, Gm04_15703616, and Gm04_46466250 are loci related to soybean grain weight per plant, and find the Gm09_20334173, Gm04_39518612 and Gm04_39518624 are loci related to 100-seed weight. After performing a reference comparison, we conducted gene annotation and identified candidate genes *Glyma.04G203400* and *Glyma.04G125600*, potentially associated with grain weight per plant in soybeans. These genes are primarily involved in protein synthesis and cell differentiation processes. The candidate gene *Glyma.09G109100*, associated with the 100-grain weight trait, was successfully annotated. The analysis revealed that the gene primarily involves enzyme activity, suggesting its potential role in regulating grain weight. These findings offer valuable insights into the mechanism of soybean yield and serve as a critical theoretical foundation and genetic resource for cultivating new soybean germplasm with high yield. These findings are of immense significance for future research endeavors to achieve high-yielding soybean varieties.

## Introduction

Soybeans, one of China’s traditional crops, have been cultivated for over 5,000 years (Zhao *et al.* 2019). They are an essential food crop not only in China but also globally. Soybeans are rich in protein and fat (Wang 2014), providing essential nutrition for humans. Soybeans, being an indispensable food crop in daily life, contain many health-beneficial ingredients such as soy isoflavones, soybean saponins, and soy phospholipids, etc. (Gao and Shi 2013); these components have high medicinal value and provide therapeutic effects for cardiovascular diseases, liver damage, tumors, and other illnesses. Soybeans significantly contribute to human production and quality of life (Liang and Li 2018). Therefore, as the population increases, society has a growing demand for soybean production to meet people’s expanding material needs.

Soybean yield is influenced by multiple factors and regulated by various traits (Diers *et al.* 2018). Among these factors, grain weight per plant and 100-grain weight are significant influencers of soybean yield, playing a crucial role (Assefa *et al.* 2019, Ravelombola *et al.* 2021). While some research findings have been published on the correlation traits of soybean grain weight per plant and 100-grain weight, there is still a need for further improvement in localizing the genetic loci associated with these traits and identifying relevant candidate genes.

Existing studies showed that the dominant effect influences the grain weight per plant, the dominant × environment interaction effect, and the epistasis × environment interaction effect across different locations. Changes in location have a minimal impact on the grain weight per plant in soybeans, and the dominant effect dominates the genetic main effect variance (Xue *et al.* 2020); this trait is complex, quantitatively inherited, and regulated by multiple genes. Based on a population of 147 recombinant inbred lines, a multi-year multipoint quantitative trait loci (QTL) localization was conducted for the grain weight per plant. They identified two QTL intervals located on different linkage clusters in different years. Four hundred and thirteen genes within the intervals were functionally annotated, and four genes related to them, *Glyma.01 G158700*, *Glyma.01 G156800*, *Glyma.01 G125400* and *Glyma.01 G147800* were screened out as candidate genes (Yu *et al.* 2018). Using 185 natural soybean populations as materials, the authors detected 20 association signals related to the grain weight per plant through genome-wide association analysis based on high-throughput single nucleotide polymorphisms (SNPs) localized using simplified genome sequencing. Among them, eight were newly discovered loci, 12 overlapped with those reported in previous studies, and nine were considered candidate genes for the grain weight per plant through analysis (Jing *et al.* 2018). Using a population of recombinant soybean inbred lines, the researchers localized 24 QTLs associated with the grain weight per plant using a combination of simple sequence repeats (SSRs) and specific length amplified fragment (SLAF) high-density maps, 10 of which were obtained through SSR genetic mapping. By organizing the localized QTL and predicting the functions of genes within the intervals, nine genes were identified as candidates for the target traits (Han *et al.* 2021).

100-grain weight is one of the components of soybean yield (Wang *et al.* 2013). As an essential breeding target for soybean, mining the genetic loci related to the 100-grain weight and elucidating their diverse characteristics in different germplasm resources not only provide a necessary basis for the targeted improvement of the 100-grain weight but also have great significance for the improvement of soybean yield (Chen *et al.* 2016). Xu *et al.* (2023) utilized a soybean population constructed from 300 recombinant inbred lines (RILs) and genotyped the population using whole-genome resequencing technology. A total of 38 QTLs related to the 100-grain weight of soybean were detected in five environments, and the subsequent analysis and detection yielded six candidate genes within four main-effect QTL regions (Xu *et al.* 2023). Based on phenotypic data from 20 different environments and genotypic data from 109,676 single nucleotide polymorphisms (SNPs), Qi *et al.* (2020) identified 118 genetic loci associated with the 100-grain weight in soybean through genome-wide association analysis. They analyzed three genes as candidate genes for the trait of 100-grain weight in soybeans in a region obtained by co-assaying multiple environments (Qi *et al.* 2020). Zhao *et al.* (2019) located 34 association signals related to soybean 100-grain weight by GWAS. Of these, 19 were newly discovered loci. The remaining 15 loci overlapped with the results of previous studies. In the associated signal regions in different environments, 237 genes near the peak SNP were considered candidates for 100-grain weight (Zhao *et al.* 2019).

This study utilized 292 soybean samples from different regions of China. Based on three consecutive years of phenotypic data for soybean grain weight per plant and 100-grain weight, a genome-wide association analysis was conducted using the population’s SNP genotype data. The significantly associated SNP loci underwent annotation and filtering to identify functional genes associated with the target traits. These findings enhance the understanding of the research mechanism for soybean yield and provide crucial theoretical support for the study of soybean grain weight per plant and 100-grain weight.

## Materials and Methods

### Test material

The test population used in this study consists of 292 soybean materials from different regions, and these materials have a wide range of genetic backgrounds, rich genetic variation, and phenotypic diversity (Supplemental Table 1). This population’s whole genome was sequenced in this study by SLAF-seq technology. SNPs were identified and filtered by comparison with the reference genome, and 641542 population SNPs were obtained after the filtering process.

### Field trials and trait investigations

The population was subjected to experimentation for three years at the experimental site of Jilin Agricultural University (43°88ʹN, 12535ʹE) in 2019, 2020, and 2021, respectively. A completely randomized block experimental design was used to plant four rows of each variety, with a row length of 4 m, a row spacing of 0.65 m, and three replications.

After the maturity stage of soybeans is completed, in each sample, 5 normally grown plants were randomly harvested in the middle region (to obliterate marginal effects).

Grain weight per plant: the weight of the seeds harvested from a single plant. The unit is g.

100-grain weight: the weight of 100 randomly selected intact kernels of each material measured after threshing. Unit is g.

### Phenotypic data analysis

Descriptive statistics, histograms of frequency distributions were constructed, analysis of variance (ANOVA), and calculations were performed on data related to grain weight per plant of soybean plants and 100-grain weight of soybean seeds using R 4.1.3 software (https://www.r-project.org/).

### Genome-wide association analysis

In this study, the population structure matrix (Q) and kinship matrix (K) were computationally estimated based on 641542 SNP loci with minor allele frequency (MAF) > 0.05 and locus integrity (INT) > 0.5 using genome-wide association analysis. A standard Bonferroni threshold of P < 1.0 × 10^–3^ (–log10 P < 3.0) was utilized to interpret significant associations between SNP loci and traits. Association analyses were performed using two analytical models, GLM (Sul and Eskin 2013) and MLM (Bradbury *et al.* 2007), with the previous Q and K matrices acting as fixed and random effects, respectively.

### Screening and analysis of candidate genes

Candidate genes were located within each significant SNP peak’s 100 kbp flanking genomic region. They were classified and annotated based on the physical location of the screened SNP markers in the Glycine_max: Wm82.a4.v1 reference genome. To predict the candidate genes related to grain weight per plant and 100-grain weight traits of soybean. GO (https://geneontology.org/) and KEGG (https://www.kegg.jp/) were used to annotate the candidate gene sets, and the functional genes related to the target traits in the localization region were predicted. The genes were preliminarily determined to be related to the characteristics of grain weight per plant and 100-grain weight, and the candidate genes were analyzed.

### Data availability

The datasets presented in this study can be found in online repositories. The sequencing data has been uploaded to NCBI (PRJNA940512).

## Results

### Analysis of phenotypic data on grain weight per plant and 100-grain weight of soybean

This study was based on 292 natural population materials composed of soybean germplasm. The population was planted in the experimental base of Jilin Agricultural University in 2019, 2020, and 2021, respectively. The yield-related traits of mature soybeans were measured, and the phenotypic data related to grain weight per plant and 100-grain weight were collected. The 3-year phenotypic data analysis results showed that the population showed significant variation in grain weight per plant and 100-grain weight, indicating that these traits were rich in genetic variation (Table 1).

**Fig. 1.**
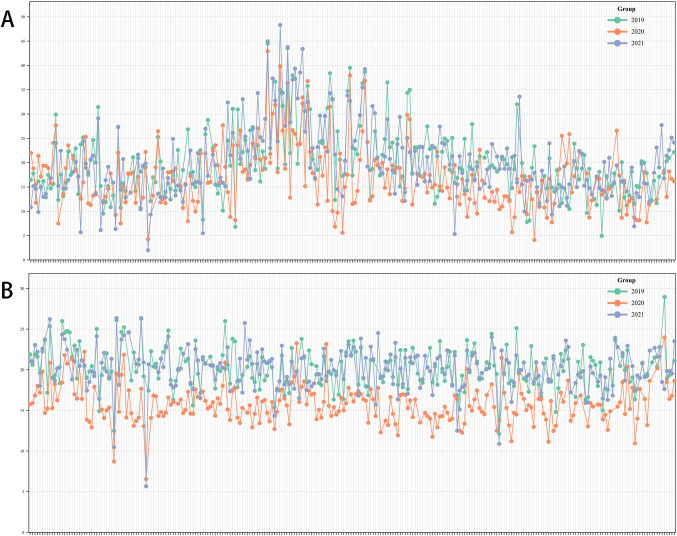
Grain weight per plant and 100-grain weight of soybean.

**Fig. 2.**
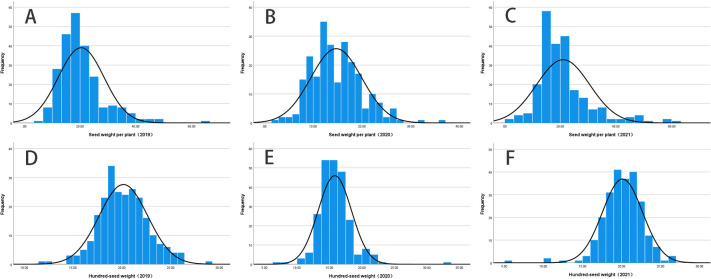
Phenotypic data distribution of soybean grain weight per plant and 100-grain weight traits in different years. (A–C) Soybeans’ grain weight per plant traits in 2019, 2020, and 2021, respectively; (D–F) The 100-grain weight traits of soybeans in 2019, 2020, and 2021, respectively.

The 3-year average grain weight per plant of the soybean population was 18.71 g, ranging from 33.56 to 60.85 g, and the coefficient of variation was 0.4, 0.36, and 0.45, respectively. The 3-year average 100-grain weight of soybean was 18.7 g, ranging from 16.84 to 26.71 g, and the coefficient of variation (CV) was 0.12, 0.16, and 0.13, respectively (Table 1, Fig. 1). The estimated heritability of grain weight per plant and 100-grain weight per plant was 84.47% and 81.87%, respectively. These values suggest that most of the phenotypic variation observed in these traits is influenced by genotype and controlled by genetic factors. Notably, the Kolmogorov-Smirnov test confirmed that the data followed a typical distribution pattern (Fig. 2).

### Genome-wide association studies on grain weight per plant and 100-grain weight of soybean

**Fig. 3.**
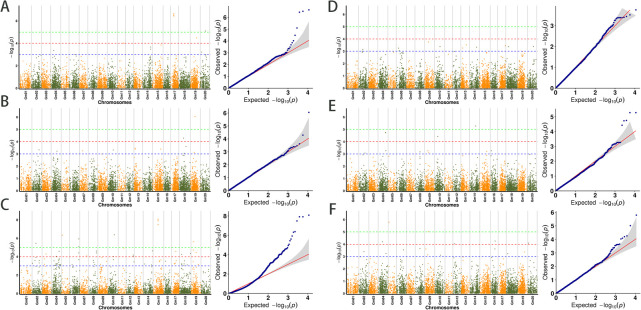
Based on the soybean phenotypic data of 2019, 2020, and 2021, it was visualized by two model analyses: the Manhattan plot and the Q-Q plot. (A–C) The Manhattan map of the grain weight per plant in the 2019, 2020, and 2021 years of the GLM model, Q-Q figure; (D–F) The Manhattan map of the 100-grain weight in the 2019, 2020, and 2021 years of the GLM model, Q-Q figure.

**Fig. 4.**
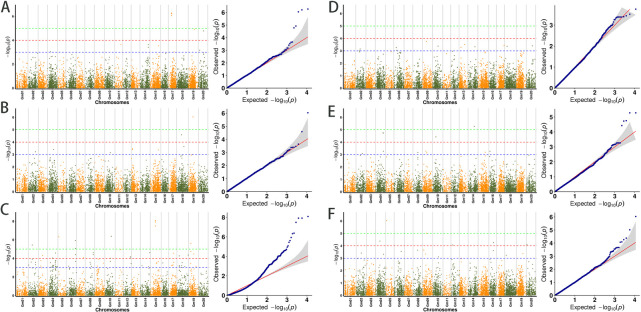
Based on the soybean phenotypic data of 2019, 2020, and 2021, it was visualized by two model analyses, the Manhattan plot and the Q-Q plot. (A–C) The Manhattan map of the MLM model with grain weight per plant in 2019, 2020, 2021, Q-Q figure; (D–F) The Manhattan map of the MLM model with 100-grain weight in 2019, 2020, 2021, Q-Q figure.

Phenotypic data on grain weight per plant and 100-grain weight of natural populations of soybeans in different environments were combined with population SNP genotype data. In order to identify significant SNP loci associated with the target traits, two GWAS analysis models (GLM, MLM) were used in this study to analyze high-quality SNP loci in the 292 soybean materials used in this study based on the critical threshold of –log10(P) < 3.0 and to complete the further screening of genetic loci associated with the target traits. To ensure the precision and genetic consistency of the mapping outcomes, we specifically chose the results simultaneously mapped by two models across a minimum of two environments. The results of the analysis of the 100-grain weight characters and the grain weight per plant are as follows (Figs. 3, 4).

**Fig. 5.**
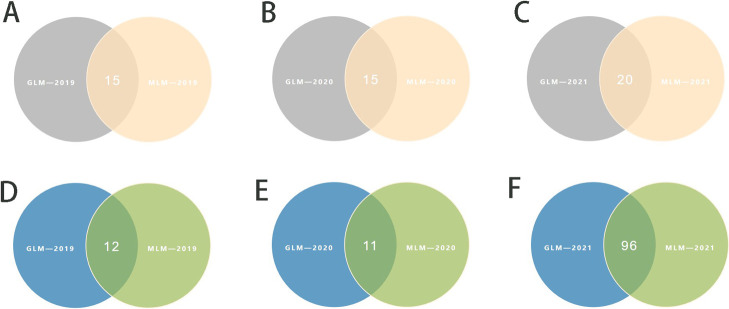
Statistics on the number of significant SNP loci related to grain weight per plant and 100-seed weight between the GLM and MLM models from 2019 to 2021. (A–C) The number of significant SNPs by 100-grain weight in soybeans co-located using GLM and MLM models in 2019, 2020, and 2021, respectively; (D–F) The number of significant SNPs grain weight per plant in soybeans co-located using GLM and MLM models in 2019, 2020, and 2021, respectively.

For the 100-grain weight trait in soybean (Fig. 3), we detected 30 significant SNP loci associated with the trait based on the GLM and MLM models in 2019. Taking the intersection of the localization results of the two models, we found that 15 were localized in the two models and that these loci were widely distributed on chromosomes Chr.02, Chr.06, Chr.09, Chr.10, Chr.13, Chr.14, Chr.17, Chr.18, and Chr.20 (Fig. 5A); in 2020, based on both analytical models, 30 significant SNP loci were detected, of which 15 loci were detected in both models and distributed on Chr.02, Chr.04, Chr.08, Chr.09, Chr.10, Chr.14, Chr.15, and Chr.16 (Fig. 5B); in 2021, 40 loci were detected by the two analyzed models, and the same SNPs that both models localized were also present, which were widely present on 12 different chromosomes, Chr.01, Chr.03, Chr.07, Chr.11, Chr.13, Chr.14, and Chr.15. The presence of trait-associated loci was not found in Chr.17 (Fig. 5C). When comparing the results, we found that three SNP loci (Gm09_20334173, Gm04_39518612, and Gm04_39518624) significantly associated with soybean 100-grain weight were all obtained from two analyzed models, which were localized in at least two years of environment. We concluded that these three SNPs are genetically stable and closely associated with 100-grain weight content in soybeans.

For the grain weight per plant (Fig. 4), 14 and 13 SNPS related to traits were detected by the GLM and MLM models, respectively, in 2019, and 12 could be located in both models. These SNPS are distributed on chromosomes Chr.04, Chr.11, Chr.14, Chr.15, Chr.17, Chr.19, and Chr.20 (Fig. 5D). In 2020, 12 and 11 loci were detected by GLM model and MLM model, respectively, and both models located 11 loci distributed in chromosome Chr.02, Chr.04, Chr.07, Chr.10, Chr.13, Chr.15, Chr.18, and Chr.19 (Fig. 5E). In 2021, the GLM and MLM models detected 192 SNP loci and 96 intersections of the two models were detected. These loci were widely distributed on 17 chromosomes, among which no related loci were found on the three chromosomes of Chr.01, Chr.13, and Chr.20 (Fig. 5F). By comparing the localization results in three years of environment, we found that different models in different environments were distributed on the three chromosomes of Chr.04, Chr.15, and Chr.19. In order to study the phenotypic effects corresponding to SNP loci significantly related to grain weight per plant in soybean, we selected significant SNP loci Gm04_21489088, Gm04_15703616 and Gm04_46466250 with the highest threshold as the target loci for the following studies.

### Candidate gene mining and analysis

Significant genetic loci were identified based on previously screened target traits in soybeans. We compared the 100 kbp flanking genome region around the peak significant SNP loci with the reference genome Glycine_max: Wm82.a4.v1, taking into account the chromosome and physical position of the SNP. Based on the location information provided by the reference genome, we categorized and annotated the candidate genes.

**Fig. 6.**
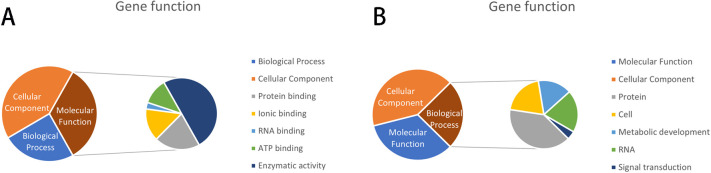
The functions of the screened genes are mainly distributed in the above three aspects. (A) The functional description of the 100-grain weight trait of soybean, and the function is mainly distributed in Molecular Function, Biological Process, and Cellular Components are not classified; (B) The functional description of grain weight per plant, and the function is mainly distributed in Biological Process, Molecular Function, and Cellular Component are not classified.

GO and KEGG annotations were performed on the genes in the 100-kb flanks of SNP loci Gm04_39518612, Gm04_39518624, and *Gm09_20334173* related to 100-grain weight. The results showed that the genes mainly concentrated in synthetic and metabolic pathways. A total of eight genes were screened for their relevance to 100-grain weight traits with diverse functional distributions (Fig. 6A). Among them, one functional gene (*Glyma.09G109100*) was annotated in the 100-kb flank of *Gm09_20334173*, which is involved in the serine/threonine kinase activity of transmembrane receptor proteins, ATP binding, and protein phosphorylation. A total of 31 genes associated with the grain weight per plant were screened and analyzed in the 100-kb flanking annotations of SNPs associated with the grain weight per plant (Gm04_21489088, Gm04_15703616, Gm04_46466250). The results showed that the gene functions were mainly focused on the aspects of protein synthesis and influencing enzyme activities (Fig. 6B). Among them, the genes *Glyma.04G203400* and *Glyma.04G125600* in the 100-kb flanks of Gm04_46466250 and Gm04_15703616 were annotated to be involved in floral development and cellular differentiation, and were related to ubiquitin-protein transferase activity, respectively. We concluded that both were correlated with soybean grain weight per plant.

Therefore, we considered *Glyma.09G109100*, *Glyma.04G203400*, and *Glyma.04G125600* as candidate genes to study target traits.

**Fig. 7.**
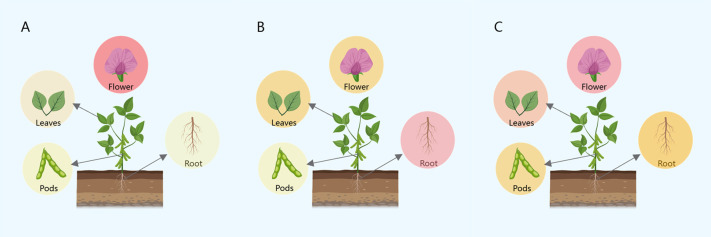
Gene expression prediction. (A) Gene *Glyma.09G109100*; (B) Gene *Glyma.04G203400*; (C) Gene *Glyma.04G125600*. Color represents the level of expression. The more vivid the color, the higher the expression. The lighter the color, the lower the term.

**Fig. 8.**
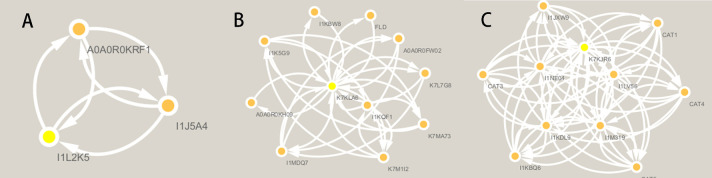
Interaction protein prediction of genes. (A) Gene *Glyma.09G109100*; (B) Gene *Glyma.04G203400*; (C) Gene *Glyma.04G125600* (PPI network constructed by STRING database. Cytoscape software further analyzes the PPl network).

This study used three genes (*Glyma.09G109100*, *Glyma.04G203400*, and *Glyma.04G125600*) as candidate genes for grain weight per plant and 100-grain weight traits. We performed expression prediction for these genes and discovered that the expression of *Glyma.09G109100*, associated with 100-grain weight in soybean plants, was mainly concentrated in the lower part of the soybeans. In contrast, the expression of this gene in the leaf, pods, and roots was more evenly distributed than in the lower part (Fig. 7A). *Glyma.04G203400*, which is associated with the grain weight per plant, showed more distribution in the roots and less expression in the flower and leaf parts compared to the roots. The gene had the slightest expression in the pods (Fig. 7B). *Glyma.04G125600* was predicted to be highly expressed in the leaf and flower parts, with a more pronounced increase in expression in the root and pod parts than the others. The expression in the root and pod parts was less pronounced than in the flower parts. Additionally, the expression at pod sites was lower (Fig. 7C). We predicted interacting proteins for the three genes and observed that *Glyma.04G125600* had relatively more interacting proteins. In contrast, *Glyma.09G109100* had the fewest predicted interacting proteins (Fig. 8).

## Discussion

Based on SLAF-seq sequencing technology (Zhang *et al.* 2022), genome-wide association analysis was conducted on grain weight per plant and 100-grain weight traits in 292 soybean germplasm resources; the data of grain weight per plant and 100-grain weight in the 3-year environment were sorted out and associated with the SNP loci related to the target traits. After screening and sorting, it was found that 3 SNPs related to soybean grain weight per plant and 3 SNPS related to soybean 100-grain weight were located on chromosomes 4 and 9, respectively. We found that in recent years, studies on the traits of grain weight per plant and 100-grain weight of soybean have been increasing. However, no QTL overlapping with the SNP loci associated with this study were found, and we speculated that differences in environmental and genetic background may have caused this (Kim *et al.* 2023). In recent years, we have found that genetic loci related to grain weight per plant, 100-grain weights, and other yield traits appear in different populations in different regions, indicating that some unique genetic loci may exist in particular environments or even in a specific material. This study selected 292 soybean germplasm with a comprehensive genetic background as test populations. The discovery of new loci may be due to differences in the natural populations constructed in this study.

Seed weight, an important index to measure soybean yield (Bheemanahalli *et al.* 2022), is a complex quantitative trait regulated by multiple genes (Luo *et al.* 2023). Wang *et al.* (2021) proposed that the seed size of a legume has an impact on its weight and that seed size is an essential factor affecting yield. The study on the regulatory mechanism of seed size and the essential genes controlling seed size is of great value for improving the yield and quality of legumes (Wang *et al.* 2021). Yu *et al.* (2023) also believed that differences in seed size were closely related to seed weight. As a significant agronomic trait in crop domestication, seed size further affected soybean yield by influencing grain weight (Yu *et al.* 2023). The candidate gene *Glyma.04G203400*, related to grain weight per plant, was located and screened in this study. GO annotation indicated that this gene involved cell differentiation and influenced flower development. Multiple factors and genes influence the size of seeds. At the cellular level, seed size depends on the synergistic effect of cell proliferation and cell expansion during seed growth and development (Hepworth and Lenhard 2014). This gene may influence seed weight by affecting cell differentiation, growth, and size. In addition, existing studies show that in 2017, Lu *et al.* (2017) cloned *J*, an essential gene for determining the long childhood traits of soybeans. The *J* gene is not only associated with flowering, but the mutant version of this gene can also delay the flowering time of soybeans in low latitudes and short days, thereby increasing the yield of soybeans by 30% to 50% compared to the control wild-type. It is conducive to the large-scale cultivation and popularization soybeans in tropical equatorial regions (Lu *et al.* 2017). This study shows that soybeans’ flowering time or mechanism can affect their yield. The candidate gene *Glyma.04G203400* may alter soybean yield by impacting cell differentiation and flowering.

The study, *Glyma.04G125600*, whose GO annotation results showed that it has many functional descriptions, among which we found that this gene has a molecular function that is ubiquitin-protein transferase activity. Several studies have shown that ubiquitination is vital in many cellular and physiological processes (Yu 2022), and ubiquitin-mediated proteolysis regulates seed size. Du *et al.* (2017) analyzed molecular components in seed coat and embryo that may contribute to differences in seed size, indicating that a gene encoding the E3 ligase zinc finger protein, *Glyma.17G202700*, is highly expressed in the seed coat of large seeds. The gene *Glyma.07G196500* for the E2 conjugated enzyme phosphate 2 is also highly expressed in the seed coat of large grains. According to the results, the ubiquitin-mediated proteolysis pathway operating at the seed coat site may impact seed size (Du *et al.* 2017). So, the gene *Glyma.04G125600* might regulate seed size by participating in the ubiquitin process. The function of the 100-grain weight related gene *Glyma.09G109100* is related to the activity of a transmembrane receptor protein serine/threonine kinase, which can regulate different cell functions and play a role in different aspects such as metabolism, growth, and transcription. Therefore, this gene may affect soybean kernel weight by influencing cell metabolic growth. Further, it changes the yield of soybeans.

## Author Contribution Statement

TS was responsible for the experiment’s design and the paper’s writing. QZ and LL were responsible for the measurement of phenotypic data. YT is responsible for the collection of receipts; JW is responsible for data analysis; KW and BY are responsible for software analysis; PW is responsible for the formal analysis and supervises the experiment.

## Supplementary Material

Supplemental Table

## Figures and Tables

**Table 1.** Descriptive statistical results of soybean grain weight traits per plant and 100-grain weight traits under three years

Traits	Years	Mean	Median	Min	Max	Skew	Kurtosis	Standard error (SE)	Coefficient of variation (CV)	H^2^
Grain weight per plant	2019	20.39	18.73	4.95	64.68	1.58	4.03	0.53	0.4	84.47%
	2020	14.73	13.97	2.8	36.36	0.68	0.84	0.34	0.36	
	2021	21.02	19.01	1.5	62.48	1.63	3.75	0.62	0.45	
100-grain weight	2019	20.16	20	12.11	28.95	0.15	0.6	0.16	0.12	81.87%
	2020	15.83	15.63	6.55	33.26	1.44	8.95	0.17	0.16	
	2021	20.13	20.26	5.67	26.37	–1.06	4.7	0.17	0.13	
